# Editorial: The interplay of stress, health, and well-being: unraveling the psychological and physiological processes, volume II

**DOI:** 10.3389/fpsyg.2025.1604339

**Published:** 2025-04-29

**Authors:** Adelinda Araújo Candeias, Edgar Galindo, Konrad Reschke, Marcus Stueck, Mariola Bidzan

**Affiliations:** ^1^University of Evora, Évora, Portugal; ^2^School of Health and Human Development, Department of Medical Sciences and Health, Comprehensive Health Research Center, University of Evora, Évora, Portugal; ^3^Comprehensive Health Research Centre, University of Evora, Évora, Portugal; ^4^Leipzig University, Leipzig, Germany; ^5^International Biocentric Research Academy (IBRA), Leipzig, Germany; ^6^University of Gdansk, Gdansk, Poland

**Keywords:** stress, health, wellbeing, resilience, mindfulness, social support

## Introduction

The increasing complexity of modern life has amplified the impact of stress on health and wellbeing, making it one of the most pressing challenges of the 21st century. Stress, whether acute or chronic, has profound effects on physical health, mental resilience, wellbeing and overall quality of life. Following the success of the first volume, this second installment of *The interplay of stress, health, and well-being: unraveling the psychological and physiological processes, volume II* continues the exploration of the dynamic relationships among these factors. By integrating insights from psychology, neuroscience, physiology, and public health, this volume presents novel research aimed at understanding the mechanisms through which stress affects individuals and how targeted interventions can mitigate its negative consequences. This volume collectively represents over 35,000 views, until the moment, demonstrating the growing academic and clinical interest in the topic.

Chronic stress is increasingly recognized as a key risk factor for numerous health conditions, including cardiovascular diseases, immune dysfunction, metabolic disorders, and mental health challenges. Several studies in this volume explore the physiological and psychological pathways through which stress contributes to these conditions. *The impact of job stress on job satisfaction and turnover intentions among bank employees during the COVID-19 pandemic* by Lin et al. highlights the consequences of occupational stress on work attitudes and mental health. Similarly, *Non-support from the immediate boss is associated with stress and unsafety at work* by Iredahl et al. reveals how leadership and workplace culture shape stress experiences. *The relationship between neuroticism as a personality trait and mindfulness skills: a scoping review* by Angarita-Osorio et al. explores the relationship between personality traits and coping strategies. Other studies delve into the physiological consequences of stress. *Osteoarthritis with depression: mapping publication status and exploring hotspots* by Zhang et al. examines the interplay between chronic pain and mental health. *Alarm fatigue and sleep quality in medical staff—a Polish-Czech-Slovak study on workplace ergonomics* by Rypicz et al. explores the impact of workplace conditions on sleep disturbances, while *Association of quality of life with marital satisfaction, stress, and anxiety in middle-aged women* by Rakhshani et al. highlights the relationship between stress and personal wellbeing. *Coping, connection appraisal, and well-being during COVID-19 in the U.S., Japan, and Mexico* by Benjamin and Wang explores cross-cultural differences in stress adaptation. *Health-promoting lifestyle behaviors and its association with sociodemographic characteristics in hospital clinical staff* by Moghimi et al. examines the intersection between occupational stress and health behavior.

Meta-analytical work such as *The relationship between risk perceptions and negative emotions in the COVID-19: a meta-analysis* by Zhou et al., provides a broader perspective on how stress manifests across different contexts. *Changes in cognition, coping, pain and emotions after 12-months access to the digital self-management program EPIO* by Strand et al. investigates digital interventions for stress management. Additionally, *Evaluation of the mental health status of intensive care unit healthcare workers at the beginning of COVID-19 pandemic* by Özgündüz et al. assesses occupational stress in frontline healthcare providers.

A key theme of this volume is the role of resilience in mitigating stress-related health risks. Studies explore factors such as mindfulness, social support, and cognitive flexibility as protective mechanisms. *Exploring the relationship between occupational stress, physical activity, and sedentary behavior using the Job-Demand-Control Model* by Clinchamps et al. highlights the importance of psychological and physical strategies in managing stress. *Global self-esteem and coping with stress by Polish students during the COVID-19 pandemic* by Kupcewicz et al. emphasizes the importance of self-esteem in stress adaptation.

In this context, mindfulness seems to play an important role in managing stress, according to several authors. *From strain to strength: a yearlong study on the transformative influence of inner engineering online program on mental well-being* by Swaminathan et al. showcases structured mindfulness-based programs fostering resilience over time. *Dispositional mindfulness profiles and psychological symptoms: a latent profile analysis* by Mehrabi and Beshai explores how mindfulness traits impact mental health. *Mindfulness and academic procrastination among Chinese adolescents: a moderated mediation model* by Yue et al. and *Effects of mindfulness on test anxiety: a meta-analysis* by Yılmazer et al. are devoted to the application of mindfulness in the academic context. Other studies provide deeper understanding of the role of mindfulness, in the control of occupational stress in teachers (Bian and Jiang), about the interactions of mindfulness, flow experience and stress (Hohnemann et al.) on the sustained impact of mindfulness (Sercekman), on the effects of mindfulness on anxiety and depression (Alvarado-García et al.) on the application of mindfulness in health promotion (Quiroz-González et al.), and finally, on the relation between mindfulness and sleep (Li et al.).

The social context plays a critical role in shaping stress responses and coping mechanisms. *Let it stay in the heart: cultural and gendered experiences of distress among Syrian refugees in Jordan* by Lambert et al. examines stress and resilience among displaced populations. *Psychosocial hazards and work-life balance: the role of workplace conflict, rivalry, and harassment in Latvia* by Paegle et al. explores workplace dynamics. *The psychological mechanism of basic psychological need frustration affecting job burnout: a qualitative study from China* by Shi provides insight into workplace motivation and employee wellbeing. *Factors associated with teachers' intention to leave their profession: teacher portraits from two European countries* (Martinsone et al.) is devoted to the relation of stress in the workplace ante the intention to leave the profession.

Other studies contribute to the understanding of additional insights: the wellbeing of spouses of ill persons (Treister-Goltzmann and Peleg), the impact of stress on academic performance (Pǎduraru et al.), the relationship between developmental violence and wellbeing (Jupudi et al.), the concept of caring stress in health personal (Goudarzian et al.), the gender-specific perception of job stressors (Heub et al.), occupational stress in nurses (Zhong et al.) and the effects of sleep quality on stress (Koike et al.).

A special mention deserves the study by Magán et al. *PsicoCare: a pilot randomized controlled trial testing a psychological intervention combining cognitive-behavioral treatment and positive psychology therapy in acute coronary syndrome patients*, presenting a successful intervention program to control stress in severe ill patients.

## Conclusion and future directions

The studies presented in this volume underscore the multifaceted nature of stress, illustrating its physiological, psychological, and social dimensions. Three major structural themes emerge: the direct impact of stress on mental and physical health, the role of resilience and coping mechanisms, and the critical influence of social and environmental determinants on wellbeing. Across these themes, the research highlights how stress operates as a universal human response, shaped by both internal factors such as personality and cognition and external determinants such as occupational conditions, cultural expectations, and societal crises.

A defining characteristic of this volume is its intercontinental and global perspective on stress. The collected studies span diverse geographical and cultural contexts, from occupational stress in China and Latvia to mindfulness interventions in India and the psychological wellbeing of refugees in Jordan. This global scope reinforces the urgency of developing integrative models that transcend local or discipline-specific explanations of stress, instead recognizing it as a shared human experience requiring comprehensive, interdisciplinary solutions. The evidence presented underscores the need for culturally sensitive approaches to stress management, acknowledging that coping strategies effective in one context may not be directly applicable to another.

Future research should continue to expand on personalized stress interventions, integrating advancements in artificial intelligence and big data analytics to refine predictive models of stress response. Longitudinal studies on the sustained effectiveness of mindfulness-based interventions, digital health applications, and policy-driven strategies for mental health promotion will be crucial in shaping public health responses. Moreover, collaboration between psychology, neuroscience, and global health fields will be essential for advancing theoretical models that account for both biological and psychosocial determinants of stress.

On a practical level, the findings in this volume can have far-reaching implications for workplace policies, educational systems, and healthcare programs. Organizations should prioritize mental wellbeing initiatives, embedding resilience training and stress-reduction programs into their institutional frameworks. Schools and universities should foster environments that support students' psychological wellbeing, integrating mindfulness and self-efficacy training into curricula. Healthcare providers and policymakers must recognize stress not just as an individual burden but as a collective challenge requiring systemic interventions.

By bringing together these diverse perspectives, this volume not only advances our understanding of stress and wellbeing but also provides a foundation for future investigations that can lead to more effective interventions and policy recommendations. As stress continues to shape human experiences worldwide, interdisciplinary, culturally informed, and scientifically rigorous approaches will be essential in ensuring that individuals and communities can navigate its challenges with resilience and adaptability.

